# Editorial: Rio, Tokyo Paralympic Games and Beyond: How to Prepare Athletes with Motor Disabilities for Peaking

**DOI:** 10.3389/fphys.2016.00497

**Published:** 2016-10-25

**Authors:** Pierre-Marie Leprêtre, Victoria L. Goosey-Tolfrey, Thomas W. J. Janssen, Claudio Perret

**Affiliations:** ^1^UFR-STAPS, Laboratoire de Recherche “Adaptations Physiologiques à L'Exercice et Réadaptation à L'Effort”, Université de Picardie Jules VerneAmiens, France; ^2^School of Sport, Exercise and Health Sciences, Peter Harrison Centre for Disability Sport, Loughborough UniversityLoughborough, UK; ^3^European Research Group in Disability SportLoughborough, UK; ^4^Amsterdam Rehabilitation Research Center, VU UniversityAmsterdam, Netherlands; ^5^Swiss Paraplegic Centre, Institute of Sports MedicineNottwil, Switzerland

**Keywords:** paralympics, performance, disability, athletes, heat

The Paralympics are the second largest sport event in the world (Gold and Gold, 2011; Perret, 2015; Paralympics—History of the Movement, 2016). This is evident with the notable 10-fold rise in competitors from Roma in 1960 to London in 2012 (400–4237) and the remarkable growth with 176 nations that competed in Rio 2016. The performance level of athletes with an impairment have improved to a point that, in the present days, sport news and world sport movements focus on the potential advantage of artificial limbs among athletes with amputations and their integration in able-bodied competitions (Burkett, 2010). However, they do not represent the totality of athletes with impairment at the Paralympic Games. Athletes with other physical impairments [visual deficit, spinal cord injury (SCI), cerebral palsy, or others] are eligible to compete. These impairments induce typical functional and physiological (e.g., cardiovascular, thermoregulatory) responses to exercise.

Within this special editorial, the study of Reina et al. offers the reader with an understanding of the functional impairment of soccer players with cerebral palsy on the agility performance. Whereas, SCI becomes the impairment focus of Currie et al. with an emphasis on cardiac function, while Perret et al. explore the respiratory responses to exercise performance. Mechanical performance analysis (Wright; Loturco et al.), the description of physiological responses according to the functional impairment (Weissland et al.) or equipment (Abel et al.), the response to training (Paulson et al.), and the relationship between laboratory and field testing responses (West et al.) are different parts of the issue that address an important aim of the IPC: *to enable Paralympic athletes to achieve sporting excellence* (International Paralympic Committee, 2016; Paralympics—History of the Movement, 2016).

Most notable to the recent Summer Paralympic Games that have been held in Beijing 2008^1^, Rio de Janeiro 2016^2^, and are to be hosted in Tokyo (in 2020)^3^, are the potentially challenging environmental conditions of high temperatures and humidity. It has been established that these environmental conditions not only influences the level of cognitive and exercise performance capacity in trained able-bodied individuals (Veneroso et al., 2015), but their health status may also be affected. Athletes with a SCI (athletes with tetraplegia or paraplegia) are likely to experience a thermoregulatory impairment under these conditions (Goosey-Tolfrey et al., 2008). Furthermore, individuals with cerebral palsy have also demonstrated higher thermal and metabolic strain than matched controls during treadmill walking in the heat (Maltais et al., 2004). Thus, hyperthermia among these athletes with impairment could alter their performance compared to their Olympic counterparts (Bhambhani, 2002). Due to the above-mentioned impairment in thermoregulatory capacity athletes with SCI or cerebral palsy may be more susceptible to hyperthermia during exercise performed in the heat. Some studies have addressed strategies to prevent the physiologic and psychological impairments in athletic performance induced by exercise performed in the heat (Goosey-Tolfrey et al., 2008). Other proposed that wheelchair athletes should follow recommendations advocated for able-bodied individuals to minimize their risks of heat stress during competition (Bhambhani, 2002). In the present issue, the two articles of Price and Girard challenge the reader to critically examine the potential beneficial effects of thermoregulatory management strategies, particularly in athletes with SCI who performed exercise in the heat.

Finally, all contributors of this special topic provide a descriptive approach of performance, and especially the preparation of athletes with a physical impairment to optimize their exercise performance. Attention has been paid to the accuracy of tools to assess physical (Willems et al.) and physiological responses (Weissland et al.) of athletes with impairment. The motto of Paralympic movement “Spirit in Motion,” is also the philosophy of the present compendium in order to present new advances and research findings in the field of applied physiology and biomechanics in Paralymipc sport.

## Author contributions

PL: Design and scripture; VG: Completion and correction; TJ: Completion and correction; CP: Completion and correction.

### Conflict of interest statement

The authors declare that the research was conducted in the absence of any commercial or financial relationships that could be construed as a potential conflict of interest.

## Footnotes

^1^Average weather in September for Beijing, China. https://weatherspark.com/averages/34097/9/Beijing-China (Accessed July 8, 2016).

^2^Average weather in September for Rio de Janeiro, Brazil. https://weatherspark.com/averages/33421/9/Rio-de-Janeiro-Brazil (Accessed July 8, 2016).

^3^Average weather in September for Ota, Japan. https://weatherspark.com/averages/33260/9/Ota-Tokyo-Japan (Accessed July 8, 2016).
